# Can Tourism Development Make Cities More Livable? Investigating 40 Cities in China

**DOI:** 10.3390/ijerph19010472

**Published:** 2022-01-01

**Authors:** Lei Kang, Zhaoping Yang, Yunxiao Dang, Wenzhong Zhang, Caicai Liu

**Affiliations:** 1 State Key Laboratory of Desert and Oasis Ecology, Xinjiang Institute of Ecology and Geography, Chinese Academy of Sciences, Urumqi 830011, China; kanglei16@mails.ucas.ac.cn; 2 University of Chinese Academy of Sciences, Beijing 100049, China; zhangwz@igsnrr.ac.cn; 3 College of Land and Urban-Rural Development, Zhejiang University of Finance and Economics, Hangzhou 310023, China; xiaoxiao187@126.com; 4 Institute of Geographic Sciences and Natural Resources Research, Chinese Academy of Sciences, Beijing 100101, China; 5 Northwest Institute of Eco-Environment and Resources, Chinese Academy of Sciences, Lanzhou 730030, China

**Keywords:** tourism, livable city, degree of satisfaction, influence factors, multilevel model, 40 cities, China

## Abstract

The field of rapid urbanization has recently paid more attention to the relationship between tourism development and liveable city construction. Previous studies have mainly focused on the experiences of tourists in tourist cities and seldom paid attention to the perceptions of local residents. Based on survey data of nearly 10,000 permanent residents in 40 key tourist cities in China, this study uses a multilevel model to quantitatively analyse the natural environment characteristics, sociocultural environment characteristics and comprehensive attraction of tourism in different tourist cities to explore their impact on urban liveability satisfaction. Results show that the developed tourist cities do not exactly correspond to the cities with a high liveability evaluation. The objective evaluation of both the natural environment and the sociocultural environment has an important influence on the liveability of cities, but the influence of the natural environment is stronger than that of the sociocultural environment. An intermediary effect exists in the subjective evaluation of the natural environment and environments for liveability perception. Simultaneously, residents’ liveability satisfaction varies according to their age, education level, annual household income and other social and economic conditions. These findings provide insights for developing countries to further improve residents’ living quality and urban construction under the condition of the rapid development of tourism.

## 1. Introduction

With the rapid development of China’s economy and the improvement of people’s living standards, people are paying more attention to their quality of life, living environment and living conditions [1]. City dwellers pay more attention to the recreational function of the city. Urbanization and tourism have become closely intertwined phenomena [2], and tourism has become an important driving force for China’s urban economic transformation. The government has spared no effort to promote tourism in China [3]. In 2018, the overall contribution of tourism to China’s urban economy has exceeded 10%. By the end of 2018, more than 30 provinces in China had made tourism a pillar or leading industry [4]. Therefore, the development of tourism is bound to have an impact on the liveability of cities.

A liveable city is an important direction in the study of urban living environments in recent years and is also an inevitable way for future urban development. The proposal of a “liveable city” provides a new goal and direction for urban development [5]. This concept advocates for improving the living environment of residents, establishing a new model of harmonious interpersonal relationships and managing healthy urban development [6]. Previous studies on liveable cities have mainly studied the evaluation indexes, spatial distribution characteristics, factors affecting liveability, residents’ knowledge of liveability and its influence on liveable cities on different scales [7,8,9]. Tourist cities are supposed to be pleasant places for tourists, but for local residents, how they feel gets little attention. The development of urban tourism can stimulate the economy so that the government invests more financially in urban construction [6,10]. It will also enrich the city’s social culture and promote the construction of a civilized city. Simultaneously, it will bring about many problems, such as overpopulation and public resource crowding. Chinese tourist cities tend to attach importance to the scale expansion of scenic spots and often neglect urban infrastructure construction, environmental pollution control and other issues [11]. Therefore, it remains a question as to how tourist cities can develop into efficient and high-level liveable cities in the future.

Analyzing the present studies, we found some deficiencies in the existing research about tourist cities and liveable cities. We mainly want to answer two questions: (1) Are tourist cities liveable for local residents? (2) How the attractiveness of a city’s natural and sociocultural environment influences residents’ perception of urban liveability. (3) Does the development of tourism truly promote the improvement of the liveability of cities? Based on data comprising nearly ten thousand questionnaire surveys in 40 key cities in China, this paper analyses the differences in residents’ satisfaction evaluation of cities’ liveability and then explores the influence of urban tourism development factors on residents’ liveability satisfaction using a multi-level model. It is expected that through the analysis of these problems, the relationship between urban tourism development and urban liveability can be effectively recognized. It also reveals the possible ways to improve the evaluation of residents’ liveability at the city level, providing a reference for the government to build a livable city.

The remainder of the paper is structured as follows. We begin with a brief review of previous studies on urban liveability and then present our theoretical framework. This is followed by a discussion of the data, model specification and results of the multilevel models. We conclude the paper with a summary of the key findings and policy implications.

## 2. Literature Review

Liveable cities have been defined and measured in a range of different ways in the literature. European and American countries studied liveable cities first. In 1996, the second United Nations conference on human settlements introduced the concept that cities should be liveable places for human beings, advocating mainly for the importance of a pleasant living environment [12]. Since then, the study of liveable cities has been gradually carried out in the fields of geography, urban planning, sociology and other fields. A series of studies have been carried out mainly concerning healthy cities, sustainable cities and cities with comprehensive development of economic and social environments [9,13]. Some authors believe that a liveable city refers to a city with strong liveability, that is, a residence with a good living and space environment, humanistic and social environment, ecological and natural environment and clean and efficient production environment [14].

Under the influence of humanism, the city gradually becomes an aggregated function integrating life, work and leisure. Urban rest function begins to be integrated into a wider range of urban spaces and industries, and the urban leisure function also becomes an important indicator of urban liveability [15]. In this context, the improvement of residents’ spiritual and cultural needs gradually promotes the “tourization” of urban residents [16]. Additionally, the development of tourism will be important for regional economic development and will play an important role in promoting the improvement of urban functions and industrial transformation [17]. Therefore, to some extent, the rise of urban tourism promotes the improvement of urban liveability.

Tourism is an important part of urban functions. As the spatial carriers of tourism development, cities are the most important tourist destinations. The empirical research has shown that excellent tourist cities are of great significance to the improvement of the quality of the tourism destination and the tourism experience environment, the promotion of the tourism brand of the marketing towns and the promotion of urban tourism as the leading modern service industry [18]. Some studies have found that there are driving forces to improve the liveability of urban spaces in the process of tourism development [19,20]. The development of tourism brings people, information and money into cities. The function of the city as a shopping, entertainment and cultural centre will be strengthened, and the motivation to improve the attractiveness of the city will promote the city itself to continuously optimize its tourism products and city image. Other scholars believe that the development of urban tourism is conducive to enriching the attraction elements of tourist cities and improving the quality of the urban tourism experience environment, which is conducive to promoting the process of harmonious and liveable urban ecological civilization [8].

However, from another point of view, the development of tourism will also have a significant negative impact on urban development. Some studies have found that once tourism development reaches a certain threshold, the tourist attraction of a city decreases. Excessive tourism development easily leads to low efficiency in urban planning, disorderly land use, unreasonable layout and uncontrolled investment. In turn, some environmental and social problems may arise, and the liveability level of the city may be adversely affected [20,21]. Haija found that if a country relies too much on tourism and regards it as the main industry pillar of economic development, it will cause certain damage to other industries, such as manufacturing and agriculture, and will also bring a series of environmental problems, including the pollution of water sources, traffic jams, population congestion, inconvenient management and so on [22]. Balaban found that a significant influx of tourists into a tourist destination in a study of South Carolina easily caused a general rise in prices and reduced the living standards of local people [23]. Therefore, how to combine the healthy and sustainable development of urban tourism with the promotion of liveable city construction has become a pressing issue for scholars, planners and the government.

Through the literature review, the academic circle has conducted many studies on both tourist and liveable cities. The relationship between tourism and the city is becoming clearer. The city is the carrier of tourist reception facilities, while tourism relies on urban development. The development of tourism has a dual impact on the development of cities. In fact, there are many factors that affect the liveability of cities, but there is still a lack of empirical research on the impact mechanism of the environmental characteristics of tourist cities on the construction of liveable cities. Existing research has focused on the experiences of tourists in tourist cities but has paid little attention to the feelings of local residents. Furthermore, the data from the research has been limited, and the existing research is mainly from social census data and relevant national statistical data, and there is a lack of inquiry from the subjective social investigation of the population. Moreover, there is no objective statistical data or integration of subjective survey data.

## 3. Theoretical Framework

The living environment level of a tourist city will directly affect the environmental quality of the city. The quality of the urban environment has an important influence on the development planning, infrastructure construction, external image, external attraction and tourism of a tourist city [24]. The tourism and rest function of a city has gradually become an important indicator affecting the liveability of a city. According to the literature review above, the development of tourism has a double effect on the liveability of cities. Geographers have paid more attention to the influence of the natural environment and sociocultural environment characteristics of tourist cities on the liveability of cities. The following will explore the impact of tourism on urban liveability perceptions from three aspects: the natural environment, sociocultural environment and tourism attraction.

The natural environment is the foundation of the human settlement environment, and the production and life of human beings and the concrete construction activities of the human settlement environment cannot be separated from the broader natural environment background [25]. A liveable urban living environment should have a natural background of open space, fresh air, clean water and a pleasant green environment. Currently, the construction of world-renowned liveable cities not only emphasizes the need for a comfortable climate and beautiful natural environment, but also pays attention to urban ecological environment protection and environmental pollution control [26]. The development of tourism cities cannot be separated from the support of the urban natural environment, which is the basis for the development of tourism. Existing studies have shown that a favourable natural environment can attract more tourists and promote the smooth development of tourism projects to achieve better economic benefits and enhance cities’ tourist attraction [27]. Therefore, the natural environmental attraction of tourist cities has an important impact on urban liveability.

The urban cultural atmosphere is an important factor influencing residents’ living quality and liveability. Mahmoudi found that the fairness of the allocation of public service facilities in the process of urban development had an impact on the liveability awareness of residents through structured observation and a questionnaire survey [28]. Other scholars believe that open and inclusive cities can improve the competitiveness and attractiveness of cities and promote the construction of harmonious and liveable cities [29]. A positive cultural atmosphere and rich cultural activities can improve the quality of the city, and can also improve the cultural taste of residents, and promote people’s physical and mental happiness, and healthy development. The historical relics and unique culture of the city are the eternal memory and spiritual home of every resident living in the city [30]. The unique historical and cultural relics and cultural environment of a city can not only improve the quality of a city, but also enhance the cohesion of a city, providing a strong spiritual power for the development and construction of a city, to build a liveable city, improve the happiness of residents, and increase the attractiveness of a city and the unique history and culture of a city [31]. The historical and cultural deposits and characteristic cultural atmosphere of the city itself can play a positive role in the living environment of urban residents.

The comprehensive attraction of urban tourism is not only the comprehensive embodiment of its natural environment and sociocultural environment, but also an important symbol of the development of urban tourism. On the one hand, urban tourism resources determine the quality of urban tourism products. Excellent tourism resources are usually the brand representatives of regional or urban tourism, and they are also key factors influencing urban tourism suitability [32]. On the other hand, the high-quality living environment and lifestyle in liveable cities have become important sources of urban tourism attraction. The existing research shows that with the construction of tourism city culture, the improvement of city appearance and the construction of environmental supervision and public service, the urban living environment can be beautified, which is beneficial to further promoting urban construction and function optimization [33].

The characteristics of the natural environment, sociocultural environment and tourism’s comprehensive attraction at the objective level of tourist cities have an important influence on the liveability of cities. However, the intermediary role of the natural environment, cultural environment and subjective evaluation of comprehensive tourism attraction in tourist cities is easy to ignore [34,35]. Relevant studies have shown that the influence of the subjective satisfaction of individual living environments on life satisfaction may be greater than that of objective physical environments [36]. Therefore, this study takes the subjective evaluation of the natural and human environments as the intermediary variable and discusses the influence of the objective environment of the tourist city on the liveability awareness of the city by acting on the subjective environmental perception and the residents.

**Figure 1:**
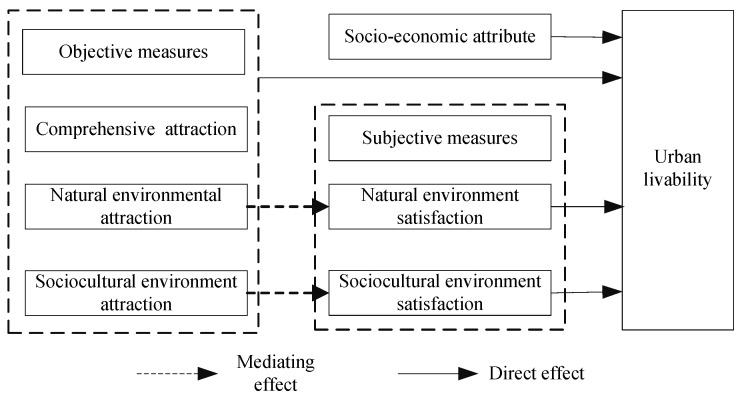
Conceptual framework of the study.

To summarize, this paper establishes the mechanism analysis framework of the influence of the subjective and objective characteristics of the natural, sociocultural and comprehensive environments on residents’ liveability evaluations in tourist cities (Figure 1). This paper first establishes the influence of objective environment, such as the effect of the natural and sociocultural environments on residents’ evaluation of city liveability, and then discusses the mediating effect of residents’ subjective satisfaction with natural and sociocultural environments. In other words, subjective satisfaction can mediate the influence of the objective environment on happiness.

## 4. Research Area and Data Sources

**Figure 2:**
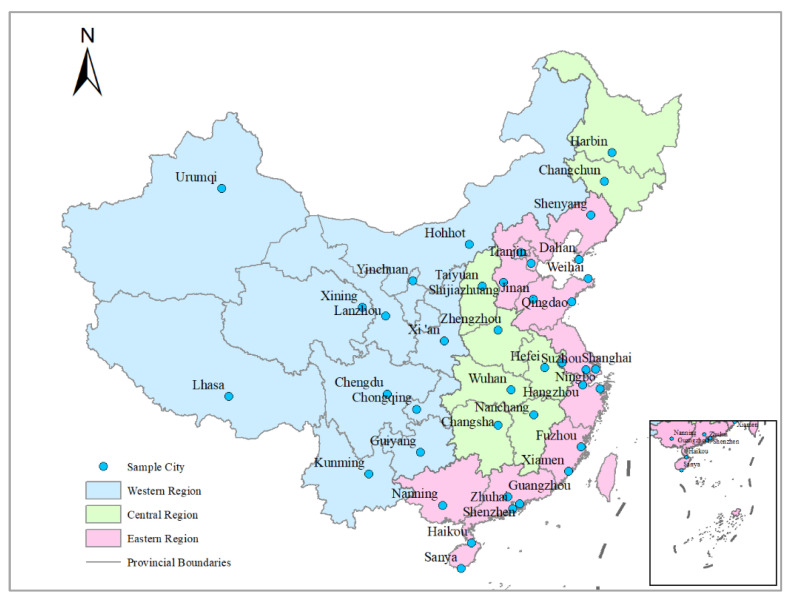
Research area. These maps were drawn according to the standard map with the drawing No. GS (2019) 1673, which was downloaded from the standard map service website of the Ministry of Natural Re-sources of the People’s Republic of China. No modifications were made on the base map.

Tourist cities refer to those that are representative, have good planning and development of tourist attractions and can reflect the development level of China’s tour-ism industry [24]. In this study, we mainly chose 40 key tourist cities in China, including Beijing, Shanghai, Guangzhou and other cities (Figure 2). By the end of 2013, China tourism administration had approved 339 outstanding tourism cities. However, due to geographical and historical factors, the development of China’s tourism industry presents a regional imbalance, with the underdeveloped areas mainly concentrated in the northwest and southwest. In order to ensure the balance between the geographical and administrative division distribution of the case cities, this study mainly selected the provincial capitals of each provincial administrative division. In addition, this paper also selected some recognized more liveable tourist cities. Considering these factors, this paper selects 40 influential tourist cities in China, taking into account not only the representativeness of tourist cities, but also the unbalanced factors of regional development. There are 40 influential tourist cities in China, including 21 in the east, 8 in the central and 11 in the west (Figure 2). It provides good material for studying an individual’s demand for different types of living environment.

The subjective data were obtained from a questionnaire survey conducted by the Research Group of Liveability City in China and comprised 40 key tourist cities in 2015. The survey participants were permanent residents who had lived in the city for more than half a year. The survey assessed 250 of the 300 municipalities directly under the central government, provincial capital and deputy provincial city according to the population size of 150–200 cities used in standard questionnaires; 12,000 questionnaires were sent out, recycling effective questionnaires, and 9,325 were returned for a questionnaire efficiency of up to 77.7%. The statistical analysis of gender, age and urban distribution of the participants showed that the samples met the control requirements, and the number and distribution structure of qualified questionnaires met the sampling design and research requirements. The statistics of individual attribute characteristics and urban characteristics of interviewees are shown in Table 1 and Table A1.

In this survey, the city’s natural environment, urban sociocultural environment, service facilities’ accessibility, transportation convenience, environmental health, safety and other important components of liveable cities were assessed. Residents’ evaluation of their satisfaction constituted the main part of the questionnaire. The questionnaire also included the socio-economic attributes of the respondents. The objective data were taken from the China Urban Statistical Yearbook [4], the official website of the National Tourism Administration, the official website of the State Administration of Cultural Relics.

## 5. Research Design

### 5.1. Methodology: The Multilevel Model

This paper simulates the impact of the urban natural environment, the cultural environment and tourism comprehensive attraction factors on residents’ liveability satisfaction at the micro-individual level and macro-city level. On this basis, attribute elements at the individual level of residents were included. When analysing such nested data, the traditional linear regression model tends to ignore the hierarchical nature of the data, which also means ignoring the role of geographical environment factors under spatial scale differentiation [35,37]. Compared with the single-layer regression model, the biggest difference of the multi-layer model is that the intercept and slope of the regression model are not fixed constants, but random variables. The remarkable advantage of the multilevel model is that it can distinguish the influence of each level of elements on the independent variables and simultaneously analyse the contribution of the level of each element to explain the differences between the independent variables. Using this model, we can better explore the influence of the natural environment, the human environment and tourism comprehensive attraction on residential satisfaction at the two levels of urban and individual residents. The model used in this study is presented below.

**Individual level:**

(1) $$Y_{jk}=\beta_{0}+\overset{m}{\underset{1}{\sum }}\beta_{m}X_{mij}+r_{ij}\;r_{ij}\sim N\left(0,\sigma_{u}^{2}\right)\;$$

**City level:**

(2) $$\beta_{0j}=\gamma_{0}+\overset{n}{\underset{1}{\sum }}\gamma_{n}W_{nj}+u_{j}\;u_{j}\sim N(0,\sigma_{e}^{2})$$

(3) $$cov\left(r_{ij},u_{j}\right)=0$$

A multilevel model was used to analyse the factors influencing the evaluation of urban liveability by residents of major tourist cities in China. The dependent variable of the multilevel regression model was the satisfaction of the residents with the perception of urban liveability. Among them, the information on residents’ liveability in their cities is the overall evaluation of the existing living conditions by residents via questionnaires (including ease of living, safety, comfort of the natural and cultural environments, travel convenience and environmental health). Responses were quantified on a 5-point Likert scale ranging from 1 (extremely dissatisfied) to 5 (extremely satisfied). This paper focuses on the factors that affect the perception of liveability in cities. The explanatory variables of the city level include natural environment, Sociocultural environment and comprehensive attraction of tourism cities. The index of the individual level is evaluated by the indexes of each dimension of the interviewees in the questionnaire.

#### 5.1.1. Natural Environmental Attraction

The attraction of the urban natural environment mainly refers to the unique advantages of cities in terms of the natural environment. In terms of objective indicators, we mainly choose the number of urban parks, urban green rate, urban air quality days and urban scenic spots to express this aspect. In terms of subjective indicators, we mainly choose climate comfort, the urban green coverage rate and comprehensive evaluation of the urban natural environment to express this aspect.

#### 5.1.2. Sociocultural Environment Attraction

The attraction of an urban sociocultural environment mainly refers to the unique advantages of a city in terms of its social and humanistic environment. In terms of objective indicators, we mainly choose the number of libraries per 100 people in the city, the number of museums in the city and the number of key cultural relic protection units in the city to express this aspect. In terms of subjective indicators, we mainly choose the characteristic cultural atmosphere of the city and the social and cultural environment of the city to express this aspect.

#### 5.1.3. Comprehensive Attraction

The comprehensive attraction of tourists is an important reflection of a city’s comprehensive strength. The existing research has often studied the tourism attraction of cities according to tourism resource endowment and other indicators of tourism economic influence [15]. According to this study, the comprehensive attraction of tourist cities not only refers to the advantages of tourist cities in natural sociocultural environments but also further advantages in the tourism industry and infrastructure. The objective index of the comprehensive attraction of tourist cities is mainly expressed by the GDP proportion of the tourist industry, the proportion of tourism practitioners and the number of scenic spots above a 4A level (Comprehensive evaluation of tourism resources by national Tourism Administration).

#### 5.1.4. Personal Features

Existing empirical studies have shown that individual characteristic factors such as the gender, age, household registration, education background, income and family population of residents have a significant impact on residents’ life satisfaction [36]. Therefore, the above individual attribute characteristics are taken as control variables in this paper. The specific variables and their definitions are shown in Table 1.

## 6. Empirical Analysis

### 6.1. Comparison of Livability Satisfaction between Cities

**Figure 3:**
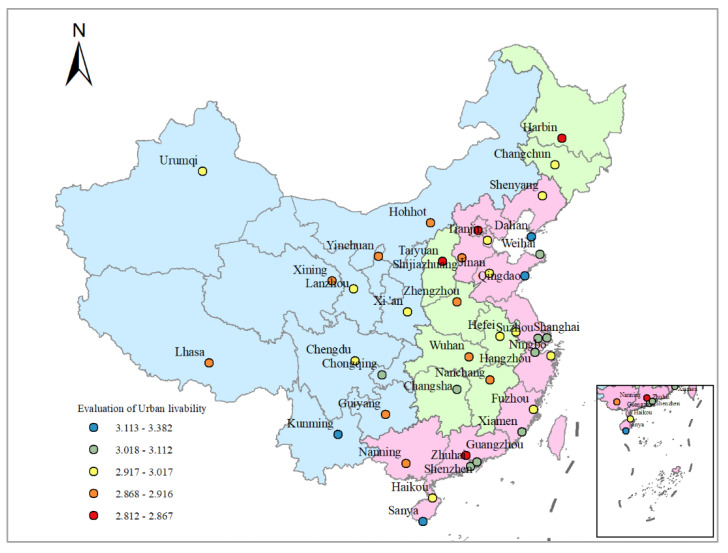
Evaluation of Urban liveability. These maps were drawn according to the standard map with the drawing No. GS (2019) 1673, which was downloaded from the standard map service website of the Ministry of Natural Re-sources of the People’s Republic of China. No modifications were made on the base map.

Liveability mainly reflects the city’s suitability for human living and living comfort. The cities with a high liveable index were mainly concentrated in the eastern coastal areas, and the cities where residents were most satisfied with their liveable cities included Qingdao, Kuming, Sanya, Dalian and Weihai (Figure 3). The high liveability index of cities in eastern coastal areas is inseparable from its high level of economic development. Secondly, coastal cities have pleasant natural environments, such as beautiful environments, fresh air, and clean and adjacent water, which are incomparable advantages of other cities. Finally, these cities have developed a tourism industry and advantages in ecological protection and environmental beautification. Their economic structure is dominated by tertiary industry. Their excellent public services and infrastructure make it easy to secure good evaluations from residents.

The cities where residents were least satisfied with urban liveability are Nanchang, Taiyuan, Harbin, Guangzhou and Beijing (Figure 3). All these cities, except Beijing and Guangzhou, belong to the central and western regions. The development of tourism lags behind; urban infrastructure is not perfect, and large-scale industrial development has created environmental pollution. Although Guangzhou and Beijing are first-tier cities with developed tourism, the large population concentration has yielded many negative impacts on the cities, such as unbalanced service facilities, housing difficulties and traffic congestion.

This section explicates that more developed tourism cities and a higher evaluation of liveable cities do not correspond to each other. Cities with high liveability evaluations are mostly cities with high levels of tourism industry development, while cities with low liveability evaluations have both cities with developed tourism and cities with backward tourism. This article will elaborate in the next section on the influence mechanism of the natural and sociocultural environments and the comprehensive attraction of tourism cities on the liveability of cities.

### 6.2. Analysis of Factors Influencing Urban Liveability

To analyse the objective characteristics of the urban natural environment, the sociocultural environment and comprehensive tourism attraction and the influence of subjective satisfaction on urban liveability satisfaction, this paper used the respondents’ liveability satisfaction as the main dependent variable and introduced individual-level variables and city-level variables into the multilevel model respectively. To compare the effects of the influencing factors, all explanatory variables were standardised before being introduced into the model. All the models studied in this paper are estimated by STATA software, and Akaike Information Criterion (AIC) is used to compare the fitting effect of different models. The smaller the value is, the higher the fitting degree of the model is [29]. In order to understand whether there are differences in the living environment of the subjective evaluation at the city scale, we use the multi-layer linear model method to extract the explicable variance proportion of the subjective evaluation at the city and resident level respectively. The results show that the variance proportion of urban and resident level is 19.3% and 80.7% respectively, indicating that there are significant differences in the subjective evaluation of urban scale, that is, residents’ evaluation of residential environment is very inconsistent among different cities.

Firstly, an empty model without introducing any individual and community variables was constructed to calculate the differences in liveability satisfaction of all samples at the city and individual levels. The chi-square value of the model was 445.38 (*P* < 0.001), indicating that the model passed the test. The LR test results show that the explanatory power of multi-layer model is significantly higher than that of the single-layer model. The influencing factors of residents’ liveability satisfaction mainly include six models (Table 2 and Table 3). Models 1–3 introduce objective variables of natural environment attraction, sociocultural environment attraction and tourism comprehensive attraction to explore their influence on liveability satisfaction. Natural environment satisfaction and sociocultural environment were added in Models 4 and 5 to examine the impacts of subjective contextual variables at different geographic scales on life satisfaction. Additionally, variables of urban location were added in Model 6. The chi-square statistics of all models were significant at the 1% level, and the Deviance Information Criterion (DIC) was within a reasonable range, indicating that the models fit well [37]. Table 3 shows that the DIC value decreased to different degrees after the introduction of the subjective satisfaction factor variables in Models 4–5, indicating that the introduction of the subjective variables of the natural environment and the social and humanistic environment had a better explanatory effect on residents’ liveability satisfaction.

The results of Model 1 show that the number of urban parks, the rate of urban greening, the urban air quality and the urban scenic spots were statistically significant for the satisfaction of residents with the attractiveness of the urban natural environment. Among the four indicators, urban air quality, a natural environment indicator, had the most significant impact on the city’s liveability. This indicates that urban air quality is the key factor affecting urban liveability.

The number of urban parks and the rate of urban greening also had a significant positive impact on urban liveability. The improvement of the greening rate is beneficial for improving the natural environment of urban residential areas and for creating a good activity space for residents. City parks not only represent the image of the city but are also a good place for residents to move and exercise. These two factors are also an important part of tourism cities’ efforts to improve the natural environment. Studies have shown that urban parks not only play an important role in maintaining the urban ecosystem and providing ecological products, but they can also meet the leisure needs of urban residents and play an important role in the construction of liveable cities [27].

However, the number of urban scenic spots had a significant negative impact on urban liveability. The natural scenic area of the city is an important destination for attracting tourists, and many urban scenic spots are of great significance to the improvement of the city’s image and the natural environment of the city. However, many visitors gather in cities, which has a negative impact on the production and life of local residents, such as rising prices, traffic congestion and environ-mental noise.

The results of model 2 show that the attractiveness of objective socio-cultural environment also has a certain influence on urban liveability. Among them, urban cultural relic protection units and urban museums have a positive impact on the satisfaction of liveability. A good social and cultural environment plays a positive role in the high-quality living environment of a city, which is conducive to residents’ positive evaluation of the social and economic development of a city, and also improves the liveability of a city. However, compared with the attractiveness of natural environment, the influence of social and cultural attraction on urban liveability is relatively low. The possible reason is that museums and other cultural service facilities in many cities in China are relatively single, and no effective cultural service product system has been formed. The natural environment may be more valued by residents.

The results of Model 3 show that the proportion of tourism GDP and the number of scenic spots above a 4A grade have a negative influence on urban liveability in the comprehensive attraction of urban tourism. This also further shows that a good tourist city is not necessarily a very liveable one. Tourism is an important driving force for urban development, and a city’s 4A and 5A scenic spots are important symbols of urban tourism attraction. However, the large-scale development of tourism has had many negative effects on the construction of liveable cities. Tourists entering destinations on a large scale not only create more pressure for the natural environment, but also lead to intensified competition in urban public spaces and even potential cultural shock and social equity problems.

Model 4 added subjective satisfaction with the natural environment to the basis of Model 1. The results showed that the significance of objective variables describing the natural environment was reduced after the introduction of subjective variables of the natural environment. The subjective variables of the natural environment comprised the overall evaluation of the natural environment and climate comfort, which were significant. This suggests that perceived natural environment is a more important predictor of urban liveability than objective variables of the natural environment and that perceived natural environment substantively mediates the relationship between the objective variables of the natural environment and urban liveability.

Model 5 added the subjective satisfaction of sociocultural environment to the basis of Model 2, and also found that the indicators of objective sociocultural environment are no longer significant, while the subjective evaluation of sociocultural environment is more significant, which indicates that subjective sociocultural environment assessment also has a mediating effect. These findings provide solid grounds for our postulation that subjective measures of satisfaction might act as mediators in the links between objective measures and the liveability of cities. In Model 6, urban location is introduced into the model as a classification variable, indicating that the groups living in eastern tourist cities are more affected by the living environment, followed by western cities and central cities. The eastern region has a humid climate and superior natural conditions. Existing studies have also shown that wetter climates near large water sources have a more significant impact on life satisfaction [38].

In terms of individual economic and social characteristics, gender, age and family income have a certain influence on residents’ liveability satisfaction, which is similar to some existing research results [39]. Compared with men, women may have higher liveability satisfaction, which indicates that men may face greater housing pressure in China. Concerning family income, with the increase of family income, residents have better economic conditions to improve living conditions, thus improving residents’ liveability satisfaction. The model shows that the influence of age on liveability satisfaction is positive. Middle-aged people with mature careers and families gradually have the ability to meet their own living needs, while the living conditions of young people in the initial stages of their careers make it difficult to meet their existing needs. Unexpectedly, the impact of household registration on residents’ liveability satisfaction was not obvious. One possible reason for this that with the expansion of urbanization, the local poor and low-income population are faced with large housing, employment and social problems, and their liveability satisfaction may be low.

## 7. Discussion

This study explored the impact of urban tourism attraction on urban liveability from the aspects of natural environment and socio-cultural environment. The natural environment is an important part of realizing the liveable function of the city. Many studies have found that the natural environment has a significant impact on residential satisfaction [40,41]. This aligns with the results of this paper, and the impact of the sociocultural environment on urban liveability is relatively limited. The reason may be that China’s current urbanization quality is not high, the city’s social and cultural service facilities are not perfect and it is difficult to meet the needs of residents.

Furthermore, subjective environmental assessment has a greater impact on urban liveability than objective environmental assessment, which is consistent with the results of previous studies [41,42]. These subjective indicators have stronger explanatory power than objective variables, and they mediate the relationship between urban liveability and objective indicators. This provides a novel way to study urban liveability in the future.

This study found that areas with strong tourist attraction and good natural and social and cultural environment are not positively correlated with urban livability, which is inconsistent with existing studies. Most studies have found that high-quality living environment is positively correlated with life satisfaction [43,44]. One reason of the mismatch between the rate of touristic cities and their low liveability may be the impossibility of them to accommodate to the increasing affluent of tourist and the lagging of infrastructure investment as a response to that rapid change.

Although based on subjective evaluation of habitability evaluation will be affected by individual differences, but this article uses the multi-layer linear model analysis, and notes that city level variance can explain the urban liveability evaluation result difference of nearly 20%, subjective data research indicates that using the habitability of city scale differences, the scale effect plays an important role. This conclusion also provides a worthy reference for similar research in the future, that is to use the idea of data stratification to solve the problem of scale difference in subjective evaluation.

This research has important policy implications. First, from the point of view of liveable cities, although tourism is an important driving force for urban development, the excessive development of tourism in cities has a greater negative effect on the whole. The tourism development level of the city should be in a moderate level, neither too low nor too high; Urban construction needs to improve the construction level and protection of regional ecological, cultural and tourism resources, and optimize the regional features and characteristics of human settlements. We will promote the transformation and upgrading of regional features such as tourist attractions, nature reserves, and famous historical and cultural cities and towns. Keeping urban tourism within an appropriate range is conducive to improving the liveability of cities and reducing the negative effects generated by tourism.

Second, compared with the cultural environment of the tourist city, the natural environment of the tourist city has a more obvious influence on the liveability of the city. The city park and the city green rate are not only important guarantees of the attraction of the tourist city but also play an important role in urban liveability. In the future, tourism cities should pay attention to the optimal layout of urban parks, improve environmental pollution and build green ecology in harmonious and liveable cities.

Finally, in the major developed cities in Europe and America, a good urban cultural atmosphere can bring citizens healthy physical and mental development, and the quality of life of urban residents is closely linked to it. The construction of urban cultural atmosphere features is an important part of the construction of liveable cities. Although the social and cultural environment of Chinese cities has little influence on urban liveability, the diversified urban cultural atmosphere will be the direction of the high-quality development of liveable cities in the future. Urban construction in the future should pay more attention to urban humanistic environment and retain the unique regional environment, cultural characteristics and architectural style of the city, which is of great significance to improve the sense of belonging and civilized quality of urban residents.

## 8. Conclusions

How the development of tourism affects the liveability of cities is controversial in academic circles. In the context of rapid urbanization, research on urban human settlements represented by liveable cities has drawn extensive attention from scholars. In the past, researchers mainly focused on urban background environmental factors and rarely considered the impact of tourism development on liveable cities. This study developed an analytical framework to investigate the effects of both objective and subjective measures on urban liveability within 40 key tourism cities in China. First, it presented the spatial distributions of diversified evaluations of the natural environment, sociocultural environment and urban liveability at the city scale in China. The study further investigated the impact of subjective living environment evaluations on urban liveability and how they mediate the relationship between objective measures and urban liveability.

Our key findings can be organised in several aspects. First, cities with good tourism development do not correspond to cities with high liveability evaluations. A city with a high-level economy and developed tourism is not the most liveable. A liveable city with a high evaluation from residents is a city that adapts to the natural environment, sociocultural environment and urban development. Second, the objective evaluation of both the natural environment and the sociocultural environment has an important impact on the liveability of cities, but the impact of the natural environment is greater than that of the social and cultural environments. Third, after adding the subjective evaluation of the natural and sociocultural environments, it was found that the objective variables of the objective environment were weakened, which indicates that the subjective evaluation of the natural and sociocultural environments is the intermediary variable of the influence of the objective living environment on urban liveability. Finally, the difference in residents’ evaluations of urban liveability relates more to the difference of individual attributes. Among them, family income, age and gender have significant influence on urban liveability.

We acknowledge that our paper inevitably has some limitations. First, the relevant environmental indicators affecting urban liveability are not comprehensive. Due to limited data, urban parks, green spaces, libraries and other public services are only considered in terms of the objective indicators of liveability, rather than the fairness of distribution. We also did not consider the impact of urban unemployment rate, education level, affordability of medical system, availability of public transport and other indicators on liveability. We found that the objective socio-cultural environment index is no longer significant, while the subjective evaluation of socio-cultural environment is more significant. This finding may also indicate that the definition of objective variables is somewhat simplified or incomplete. Second, this paper mainly discusses the influence of urban tourism development on liveable cities but does not discuss the influence of liveable city construction on tourism city development. This will be an important direction for further research in the future.

## Figures and Tables

**Table 1:** Descriptive statistics of variables.

Variable Properties	Explanation and Coding Scheme	Mean	Standard Deviation
Independent variable (objective)			
Natural environmental attraction	City parks: Number of city parks in the city	114.95	154.03
	Urban greening rate: Urban greening rate in the city	39.32	5.32
	Air quality of cities: The number of days the air quality of cities reaches the standard	269.00	54.88
	Natural scenic spots: The number of natural scenic spots in the city	10.50	6.02
Sociocultural environment attraction	City library: The number of libraries per 100 people in the city	210.23	164.74
	City museums: Number of city museums in the city	39.49	26.11
	Culture heritage: The number of key cultural relics under protection	7.56	5.31
Tourist attraction	Tourism: Tourism as a share of GDP	22.99	19.27
	Tourism employees: The proportion of tourism employees	24.78	14.22
	Scenic spots: The number of scenic spots above 4A level	18.26	9.77
Independent variable (subjective)			
Mediated Variable	Climatic comfort: Residents’ evaluation of climate comfort: 1–5	3.06	1.34
	Afforestation coverage in the city: Residents’ evaluation of urban green coverage rate: 1–5	3.05	1.33
	Assessment of the natural environment: Residents’ evaluation of urban natural environment: 1–5	3.07	0.91
	Evaluation of urban cultural atmosphere: Residents’ evaluation of urban characteristic cultural atmosphere: 1–5	3.08	1.32
	Sociocultural environment evaluation: Residents’ evaluation of urban social and cultural environment: 1–5	3.05	0.87
control variable	Rent and purchase of a house: 1 = Rent; 2 = No rent	1.61	0.64
	Living space: Respondents’ housing area(m^3^)	72.87	32.11
	Age: 1 = (1,20]; 2 = (20,30]; 3 = (30,40]; 4 = (40,55]; 5 = (55,60]; 6 = (60,70]; 7 = 70 above	3.38	1.48
	Gender:1 = male;2 = female	1.48	0.50
	Education: 1 = high school or below; 2 = college degree or above	1.46	1.50
	Census register: 1 = Nonlocal census register; 2 = Locality census register	1.35	0.48
	Family income (RMB): 1 = [0, 3000]; 2 = (3000, 5000]; 3 = (5000, 10,000]; 4 = (10,000, 15,000]; 5 = (15,000, 20,000]; 6 = (20,000, 30,000]; 7 = 30,000 above	3.17	1.39
	Family size: The number of family members	3.10	1.18

Note: 1–5 means degree of satisfaction (1 = extremely dissatisfied; 2 = relatively dissatisfied; 3 = neutrally satisfied; 4 = relatively satisfied; 5 = extremely satisfied).

**Table 2:** Model estimation results with socio-demographics and objective variables.

Variable	Model 1	Standard Error	Model 2	Standard Error	Model 3	Standard Error
Objective Variable						
City parks	0.016 **	0.014				
Urban greening rate	0.017 **	0.013				
Air quality of cities	0.034 ***	0.015				
Natural scenic spots	−0.015 *	0.016				
City library			0.008	0.009		
City museums			0.009 *	0.014		
Culture heritage			0.035 *	0.021		
Tourism					−0.008 *	0.02
Tourism employees					-0.022	0.013
Scenic spots					−0.014 *	0.017
Control variable						
Rent and purchase of a house (Rent #)	0.003 *	0.014	0.004 *	0.014	0.003 *	0.014
Age	0.003 **	0.012	0.005 **	0.002	0.005 **	0.002
Gender (male #)	−0.051 **	0.014	−0.034 **	0.014	−0.038 **	0.014
Education (Low education #)	0.005 *	0.002	−0.020 **	0.009	−0.021 **	0.009
Census register (local #)	−0.058	0.01	−0.058	0.01	−0.058	0.01
Family income	0.022**	0.009	0.007 **	0.004	0.007 **	0.004
Family size	0.019 **	0.01	0.018 **	0.01	0.018 **	0.01
Constant	0.287	0.019	0.283	0.02	0.285	0.02
AIC	75,379.714		75,370.853		75,046.876	
DIC	11,534.783		11,524.664		11,528.111	
Chi-square	133.987		128.504		140.949	

Notes: * significant at 5%; ** significant at 1%; *** significant at 0.5%. # indicates the reference variable.

**Table 3:** Model estimation results with objective variables and subjective variables.

Variable	Model 4	Standard Error	Model 5	Standard Error	Model 6	Standard Error
Objective variable						
City parks	0.017	0.016				
Urban greening rate	0.021	0.012				
Air quality of cities	0.023 *	0.013				
Natural scenic spots	0.007	0.016				
City library			0.031	0.009		
City museums			0.027 *	0.011		
Culture heritage			−0.004	0.015		
Tourism						
Tourism employees						
Scenic spots						
Subjective variable						
Climatic comfort	0.025 *	0.012				
Urban green coverage	0.032	0.01				
Assessment of the natural environment	0.195 **	0.014				
Evaluation of urban cultural atmosphere			0.080	0.009		
Sociocultural environment evaluation			0.057	0.009		
Rent and purchase of a house (Rent #)	0.006 **	0.013	0.025 *	0.017	0.048 **	0.012
Age	0.006 **	0.002	0.009 **	0.013	0.006 **	0.002
Gender (male #)	0.048 *	0.013	0.006 ***	0.002	0.006 ***	0.013
Education (Low education #)	0.023 ***	0.008	0.009 *	0.013	0.022 **	0.008
Census register (local #)	−0.062	0.009	−0.005	0.011	−0.061	0.009
Family income	0.006 **	0.004	0.004 **	0.004	0.006 **	0.004
Family size	0.02	0.009	−0.008	0.011	0.018	0.009
Location (# Central)						
Location (West)					0.014 **	0.017
Location (East)					0.020 **	0.017
Constant	0.29	0.018	−0.003	0.017	0.272	0.02
AIC	75,021.482		75,139.272		75,012.329	
DIC	10,996.143		10,876.642		10924.852	
Chi-square	130.392		111.25		128.466	

Notes: * significant at 5%; ** significant at 1%; *** significant at 0.5%. # indicates the reference variable.

## Data Availability

Not applicable.

## Appendix A

**Table A1:** Socioeconomic characteristics of the study population.

Attributes	Variables	Sample Size	Percentage
Hukou status	Non-local	3283	35.20%
	Local	6042	64.80%
Age	<20	997	10.70%
	20–29	1734	18.60%
	30–39	2526	27.10%
	40–49	1856	19.90%
	50–59	1146	12.30%
	≥60	1066	11.40%
Gender	Female	4507	48.30%
	Male	4818	51.70%
Education	Middle school and below	1046	11.20%
	High school	1720	18.40%
	College	2261	24.20%
	Undergraduate	2975	31.90%
	Master and above	1323	14.20%
Family’ month income (RMB)	<3000	1139	12.20%
	3000–4999	1826	19.60%
	5000–9999	2703	29.00%
	10,000–15,000	2392	25.70%
	20,000–30,000	484	5.20%
	>30,000	164	1.80%
location	Eastern region	4675	50.10%
	Central region	1835	19.70%
	Western region	2815	30.20%
